# Coaching Home Care Clients to Prepare Their Homes for Safe Care Visits: A Mixed-Methods Study to Evaluate a Nurse-Led Educational Intervention Process

**DOI:** 10.3390/ijerph21030360

**Published:** 2024-03-18

**Authors:** Pia K. Markkanen, Rebecca J. Gore, Susan R. Sama, John E. Lindberg, Catherine J. Galligan, Margaret M. Quinn

**Affiliations:** 1 Safe Home Care Project, Department of Public Health, Zuckerberg College of Health Sciences, University of Massachusetts Lowell (UMASS Lowell), Lowell, MA 01854, USA; rebecca_gore@uml.edu (R.J.G.); susan_sama@uml.edu (S.R.S.); john_lindberg@uml.edu (J.E.L.); catherine_galligan@uml.edu (C.J.G.); margaret_quinn@uml.edu (M.M.Q.); 2 Department of Biomedical Engineering, Francis College of Engineering, University of Massachusetts Lowell (UMASS Lowell), Lowell, MA 01854, USA

**Keywords:** home care, safety coaching, process evaluation, intervention study, client safety, patient safety, caregiver safety

## Abstract

Assuring home care (HC) workers’ safety is challenging because the work environment is a private home. This paper presents the process evaluation for a proof-of-concept safety intervention study to assess whether nurse-led safety coaching, using motivational interviewing and a safety handbook, could enable HC clients to improve safety in their homes. The process evaluation objectives were to (i) document the intervention’s implementation progress and (ii) assess the intervention’s dose delivery, dose reception, and fidelity. Five agencies employing liaisons (*n* = 5) and nurse managers (NMs, *n* = 8) implemented this study’s intervention and control arms. NMs assigned to the intervention arm (*n* = 6) coached 34 clients. Process evaluation metrics were assessed with mixed-methods data from (i) surveys completed by NMs during the intervention, (ii) postintervention audio-recorded and transcribed interviews (*n* = 6) with NMs and liaisons, and (iii) study progress tracking tools. The delivered dose efficiency was 85%, measured by the distribution of safety handbook copies to clients. About 94% of clients (*n* = 32) were considered “engaged” or “maybe engaged” during the safety coaching. Most coached clients (*n* = 30) were reachable for follow-up by NMs to assess intervention progress. Despite challenges, the intervention was implemented with good fidelity. Safety coaching can be applied in many HC contexts in larger populations.

## 1. Introduction

Many older adults and people living with disabilities rely on health and personal care to live independently in their homes. Aides comprise the largest part of the paid home-based caregiving workforce, with occupational titles such as home care (HC) aide, home health aide, personal care aide, personal care attendant, homemaker, and others. While there are differences in job tasks among occupational titles, usually related to the degree of medical support provided, there is also considerable overlap with respect to tasks that present safety and health risks. Most aides assist individuals in their homes with Activities of Daily Living (ADLs) [1], such as bathing, dressing, eating, caring for incontinence, and transferring to and from a bed or chair; they also assist with Instrumental Activities of Daily Living (IADLs) [1], including household work such as cleaning and disinfecting, laundry, and food preparation. In this paper, the term “HC aide” refers to all aide occupational titles. HC aides may be employed by a private business, called an “agency”, by a publicly funded HC provider, “agency”, or hired directly by the care recipients or their family. In the USA, HC recipients are called patients, clients, or consumers; in this paper, “client” refers to all HC recipients.

In 2022, there were over 3.7 million home health and personal care aides [2]. During 2022–2032, these jobs are projected to experience the highest increases in any U.S. occupation: 804,600 jobs in total [2,3]. The growing population of older adults is driving this trend. The number of adults aged 85 and older will almost triple—from 6.7 million to 19 million—between 2020 and 2060 [4]. Most prefer to receive care in their homes, rather than moving to an institution.

Despite the very rapid job growth projected for aides, there is an urgent HC workforce shortage [5]. During the COVID-19 pandemic, the HC aide shortage worsened drastically [6]. The American Association of Retired Persons reported the situation as “the worst ever”, and described situations where older adults were struggling to cope without bathing or showering in states like Maine, Wisconsin, and Pennsylvania [7]. Since 2023, post-pandemic, the situation has not improved. In July 2023, a local Massachusetts newspaper called the workforce shortage “a never-ending crisis” and reported that new HC clients may wait up to a year for an agency-employed caregiver [8].

Although the wages for HC aides have increased somewhat during recent years, their earnings are still low nationwide. The median 2022 pay for HC aides was slightly over USD 30,000 a year and USD 14.50 an hour [2]. Nonetheless, there have been testimonies indicating that “the money is not necessarily the answer” for the caregiver shortage—some HC agencies have even offered bonuses and still struggle to recruit caregivers [7].

Improvements in work safety, job satisfaction, and well-being have been raised as potential strategies to better retain caregivers and recruit new employees [9,10,11,12,13,14], and physical and psychological safety in the home–work environment supports improved the job satisfaction of aides [15].

During the past two decades, researchers have characterized occupational safety and health (OSH) hazards among HC workers [14,16]. The OSH hazards and adverse health outcomes in HC include, but are not limited to, overexertion and other musculoskeletal injuries [17,18,19]; safety hazards, such as slips, trips, and falls [17,19,20,21]; exposure to secondhand smoke [22]; exposure to hazardous chemicals, such as cleaning and disinfecting products [23]; stress [12,24,25,26], burnout [27,28,29]; violence [19,30,31]; and bloodborne pathogen exposures [32,33]. To date, safety-related interventions typically involve education for managers [34], and training following the NIOSH Total Worker Health model for aides [35,36]. Few intervention studies have attempted to improve both HC client and caregiver safety [15,33,37,38,39].

Since 2004, the Safe Home Care Project at the University of Massachusetts Lowell, USA, has conducted research to promote the safety, health, and well-being of the HC workforce by engaging a wide range of HC stakeholders, including the Massachusetts state government, private HC and elder service agencies, HC trade associations, and labor unions. The research is based on the premise that the safety of HC workers and clients is closely linked. There are challenges to assuring safety and health in the home–work environment compared with facility-based healthcare because the physical organization of homes is highly variable and homes cannot be standardized, organized, or controlled as reliably as facility-based workplaces [14].

To address these challenges, we developed an intervention to support HC clients to improve safety conditions in their homes and designed a two-arm (intervention and control) proof-of-concept study, the Safe Home Care Intervention Study, with three phases:

(i) qualitative intervention development; (ii) implementation and effectiveness evaluation conducted in partnership with five HC agencies (the “field study”); and (iii) postintervention qualitative phase. The process evaluation described here focused on the latter two phases of the overall study.

### 1.1. Study Objectives, Research Questions, and Hypotheses

The objective of the overall intervention project was to assess whether the safety coaching intervention could motivate HC clients to improve safety conditions in their homes. The guiding research question was as follows: To what extent can HC agency-led safety coaching engage and motivate HC clients to create safer conditions in their homes? We hypothesized that clients would improve safety conditions in their homes if coached by NMs applying the intervention during the clients’ HC intake assessment.

In addition to effectiveness evaluation, public health intervention studies also include a process evaluation to determine whether an intervention was implemented as intended and lessons learned for the future [40,41]. The goal of the process evaluation described here was to inform the intervention implementation progress by documenting strengths and challenges reported by those conducting the HC client safety coaching. The specific objectives were to assess four intervention process evaluation elements: (i) reach—the proportion of the priority population with the opportunity to participate in the intervention; (ii) intervention dose delivered—type, amount, frequency of intervention activities undertaken, and key materials distributed; (iii) intervention dose received—the extent to which the study beneficiaries engaged with safety coaching activities and materials; and (iv) fidelity—the extent to which the intervention was delivered as planned. Additionally, the process evaluation assessed how easy/difficult it was for NMs to implement the intervention and whether they saw evidence that intervention led to safety improvements or had the potential to do so. The research questions for this process evaluation study were as follows: To what extent can HC clients engage and respond positively to safety coaching? What evidence will the NMs find regarding the intervention’s strengths and challenges in terms of the potential to empower clients to improve home safety conditions? We hypothesized that the implementation process evaluation metrics would provide useful information regarding how HC clients engaged and responded to safety coaching. Given that the HC industry is low-resourced, especially during the COVID-19 pandemic, we were not sure whether the NMs would find it feasible to incorporate the intervention into their client intake assessment.

The findings presented here are based on the primary data from (i) field study surveys completed by NMs during the intervention implementation; (ii) post-field-study in-depth interviews with NMs and agency liaisons; and (iii) data tracking tools applied to monitor the fieldwork progress by the research team. This paper adds to the literature by demonstrating an innovative intervention approach that is ultimately meant to improve the safety of HC aides, other home-based caregivers, and HC clients and their family members.

### 1.2. Guiding Theoretical and Methodological Frameworks

The principal guiding framework for the overall intervention study was the OSH Hierarchy of Controls model [42], which prioritizes eliminating workplace hazards and exposures at their source. The intervention was implemented by employers (i.e., HC agencies) to reduce hazards in their employees’ work environment (i.e., the HC clients’ homes).

The transtheoretical model of health behavior change [43,44] was the second guiding study framework. The overall study aimed to motivate the client to create a safer home–work environment.

Finally, grounded theory—a commonly used qualitative method to generate theory from the field data [45,46]—guided the analysis of the in-depth interviews of this study.

## 2. Materials and Methods

The University of Massachusetts Lowell Institutional Review Board approved the overall study, including the intervention process evaluation presented here (protocol of #19-112-QUI-XPD).

### 2.1. Overall Safe Home Care Intervention Project Methodology

The overall Safe Home Care Intervention Project applied an exploratory sequential design [47] as its mixed-methods approach in three different study phases. The first phase (during 2019–2021) consisted of a formative qualitative study [6,15] that informed the development of materials and methods for the second phase: the safety coaching intervention field study (during 2022–2023) [39]. The second phase applied both quantitative and qualitative approaches. The third phase (in 2023) consisted of postintervention qualitative interview sessions that interpreted the findings of the second phase. The intervention process evaluation—the focus of this paper—was conducted during the second and third phases of the overall project.

The Safe Home Care research team has applied similar three-phase exploratory sequential mixed-method designs in its earlier research projects during 2004–2018 because the three distinct phases facilitate research-to-practice (r2p) [48] methods by involving its research partners and stakeholders in developing the research instruments, as well as translating the findings into safety improvements for HC practice and policy recommendations.

### 2.2. The Proof-of-Concept Intervention Field Study

This study was a proof-of-concept work to evaluate the intervention’s potential for wider application in HC industry practice. All study participants (NMs, agency liaisons, and HC aides) were 18 years or older and provided signed informed consent.

The research team did not recruit HC clients to participate in the study—they were selected for safety coaching directly by the agencies, and identifying information was not disclosed to the research team. Agencies used three criteria for client selection: (1) clients were new to the agency, (2) client’s health condition allowed the study participation without unreasonable burden to the client, and (3) clients would stay with the agency for at least 6 weeks to allow full study participation. If agencies could not select new clients, then existing clients who had not received safety training recently were selected. Most clients were selected by the order in which they were newly assigned to NMs.

Mixed methods were conducted according to a 2-arm intervention study design with the client’s home as the unit of analysis. Three HC agencies and two elder service agencies participated in the study, including agency study liaisons (*n* = 5) who recruited NMs (*n* = 8). NMs—often in collaboration with liaisons—recruited 35 clients. In the intervention arm, NMs employed by HC agencies and elder service agencies used Motivational Interviewing (MI) techniques [49,50] to coach their clients on making safety improvements in their homes. The coaching was facilitated by a printed safety handbook and companion video. The handbook includes the following ten illustrated sections [51]: (1) preparing your home for HC, (2) the first HC visit, (3) what HC aide needs from you, (4) getting the most out of your HC visit, (5) safe HC during COVID-19, (6) how to manage pets for an aide’s HC visit, (7) what your HC aide can and cannot do, (8) household safety tips, (9) household safety worksheet, and (10) COVID-19 checklist.

NMs in the control arm performed their client intake assessment with no changes in usual practices.

Intervention effectiveness was assessed by NMs and by aides. Intervention-arm NMs completed an online baseline survey after client coaching and a follow-up survey two weeks into the delivery of HC services by aides. NM surveys assessed clients’ receptivity to coaching, barriers to making safety changes, clients’ readiness for making changes, and whether specific home safety conditions improved. Aides, who were blind to the intervention itself and the intervention status of their clients, completed safety checklist surveys to record hazards in both the control and intervention homes [39]. The intervention was developed, implemented, and evaluated in 2019–2023; the data for the proof-of-concept intervention implementation and effectiveness evaluation field study were collected in July 2022–January 2023.

All agency study liaisons and participating NMs received study materials and resource binders prepared by the Safe Home Care research team.

### 2.3. Post-Field-Study In-Depth Interviews

During April–July 2023, six post-field-study interview sessions were conducted with (i) NMs (*n* = 7) who had participated in the earlier field study, as well as (ii) agency liaisons (*n* = 4). All six interview sessions were conducted in person by one interviewer from the research team. To allow efficiency and promote information sharing, the protocol allowed multiple participants from the same agency to participate in an interview session. The agencies decided whether joint or individual interview sessions were conducted. Three sessions had two participants, two sessions had one participant, and one session had three participants.

The research team prepared an interview guide that was followed in each session (see Appendix A for examples of questions asked). All sessions were audio recorded and transcribed verbatim by a transcription service. The accuracy of each typed transcript was verified using the recording, corrected if necessary, and sent to the session participants for a second review. The corrected transcripts were coded both inductively and deductively into hierarchical themes using the NVIVO 14 Qualitative Software. The analysis of interview transcripts was guided by grounded theory [45,46].

### 2.4. Process Evaluation Elements

The public-health-related program evaluation literature provides a number of process evaluation elements [40,41]. This study focused on four elements frequently reported in other safety- or health-promotion-related intervention studies [52,53,54,55]: (i) reach—the proportion of the priority population with the opportunity to participate in the intervention; (ii) dose delivered—type, amount, frequency of intervention activities undertaken, and key materials distributed; (iii) dose received—the extent to which the study beneficiaries engaged with safety coaching activities and materials; and (iv) fidelity—the extent to which the intervention was delivered as planned.

#### 2.4.1. Reach

Reach was assessed by the number of participating agencies and their clients engaged in the study and estimating their proportion in the total number of HC clients and elder service agencies in Massachusetts.

#### 2.4.2. Dose Delivered

The dose delivered included multiple activities, resources, and materials, both at the agency and HC client levels. The safety handbook, entitled Preparing Your Home for Safe Home Care [51], was the most important material resource of this study. We used the percentage of the safety handbook hard copies distributed to new HC clients out of the total copies distributed to agencies as a proxy measure to evaluate the key “dose delivered”.

Before safety coaching, intervention-arm NMs (*n* = 6) completed an online training course called Motivational Interviewing to Improve Safety in Home Care [56] (henceforth MI-training) that focused on coaching HC clients with MI techniques to prepare their homes and themselves for caregiving visits. The research team designed the MI-training. Upon completion of this MI-training course, the participants earned 1 continuing education unit (CEU).

The NMs could also take an optional training course called Practicing Occupational Safety and Health in Home Care [57]. The course was designed to facilitate the identification and remediation of hazards in client homes.

In addition to the safety handbook delivered to each intervention client, the “safety coaching packet” also included (1) a 4 min companion video [58] summarizing the handbook, which NMs could show to clients during safety coaching; (2) the online MI-training course; and (3) the resource binder providing instructions and additional tools for safety coaching and follow-up communications. Before the start of the field study at each agency, a research team member met with the agency liaison, reviewed the study protocols, and delivered an adequate number of handbooks, resource binders, and other needed supplies.

#### 2.4.3. Dose Received

The NM intervention safety coaching included (1) a baseline session for a new HC client on preparing their homes for safe HC and (2) a follow-up session with the client no sooner than 4 days later. The assessment of the dose received was based on the online survey responses, as well as on postintervention in-depth interview findings on client engagement during the safety coaching phases and sustained interest at the follow-ups.

#### 2.4.4. Fidelity

The assessment of fidelity was also based on the safety coaching baseline and follow-up survey responses and postintervention interview findings. In addition, continuous email and phone communications between the research team and participating agencies assisted in ensuring that this study was implemented as planned, including the tracking of the intervention implementation and any challenges that NMs encountered.

## 3. Results

### 3.1. Characteristics of Participants

Table 1 presents the demographic characteristics of NMs and agency liaisons who participated in the field study and returned their demographic survey information (*n* = 12). NMs and liaisons who reported demographic information averaged 44 years of age and 92% (*n* = 11) were White females, and one reported as a mixed-race male. We did not collect any identifying information on the 35 HC clients who received safety coaching. The majority of coached clients (*n* = 35) received home health aide services, homemaking services, or personal care services [59].

The eleven participants in the six post-field-study interview sessions—NMs (*n* = 7) and agency liaisons (*n* = 4)—were all White.

### 3.2. Reach

The reach of this intervention study’s key beneficiary group included a total of 35 HC clients through 2 elder service agencies and 3 HC provider agencies. The study’s client reach represents about 0.05% of the Massachusetts priority population who need assistance with ADLs or IADLs, estimated at 65,000 in Massachusetts in 2022 [60]. In terms of the aging services network of Massachusetts, this study reached 2 out of 24 Aging Service Access Points (about 8%).

### 3.3. Dose Delivered

The dose delivered comprised multiple key intervention activities and resource components (Table 2) facilitated by the five participating agencies. For this study, the percentage of the safety handbooks distributed to HC clients served as a proxy measure to assess the key dose delivered.

Table 2 shows that the delivered dose efficiency was 85% across all agencies, as measured by the distribution of the safety handbook copies to HC clients. Two agencies delivered the handbooks with 100% efficiency. All agencies achieved at least 63% efficiency.

### 3.4. Dose Received: Clients’ Engagement during the Safety Coaching Baseline and Follow-Up Visits

Six NMs at five different agencies attempted 35 different safety coaching visits at clients’ homes. The NMs were able to coach clients in 34 homes (97%). One client respectfully declined coaching due to recent health challenges.

Table 3 presents different engagement types that NMs applied during the safety coaching baseline visit. In all visits, NMs were able to show the client the safety handbook hardcopy and leave it with the client or their family member. NMs were able to discuss sections of the safety handbook with the majority of clients (94%) using MI techniques.

Table 3 also shows that the companion video was used in one coaching visit only. During the in-depth interviews, two NMs reported that the safety handbook hardcopy enabled better conversational engagement with the client than watching the video:

*Mine was more conversation instead of looking at the book. I mean we didn’t read every single word, but we would go through it, and I think it was more of a conversation than if I had them watch the video. So, that’s why I chose that.*

- An NM at the interview session #5

*… it was easier in the home to use the [Hand]book, because it’s something that they can actually hold [then] turn the pages and look… I didn’t feel that their attention was going to be focused on a video within the home.*

- An NM at the interview session #1

One NM liked the video; however, showing it to the client required a tablet or larger display than a smartphone. She described it as follows:

*I really liked the video…the one challenge with the video is that we don’t have tablets. If it was, if this was being given to an agency that had tablets… that would be very helpful, because it would be a larger screen… Because a lot of our questions, when we go out to do the assessment, is speaking to them verbally. So I think it was helpful to have the visual from the booklet, and then the video… it didn’t load in time… when we realized we couldn’t figure it out on the phone… because the screen is so little on the phone we were using, some of [clients], it was just too small for them… if we had a tablet, it would have been utilized more.*

- An NM at the interview session #3

NMs were able to complete safety coaching follow-ups with 30 clients (88%). Out of four clients who were unavailable for follow-up, two were completely out-of-reach. Two other clients were either resistant or too ill. Almost three-quarters of the safety coaching follow-up communications were conducted by phone (*n* = 22) and one-quarter by in-person visits (*n* = 8).

Table 4 shows the clients’ engagement levels during the safety coaching baseline visits and clients’ sustained interests during the follow-up communication. NMs reported that 94% of clients (*n* = 32) were interested/maybe interested, i.e., engaged, during the baseline safety coaching process; and 83% of clients (*n* = 25) were interested/maybe interested, i.e., showed sustained interest, in implementing safety changes at follow-up. A test of proportions did not find a statistically significant difference (*p* = 0.16) between the baseline and follow-up groups.

When following up with clients, NMs reported that two-thirds of the clients had either implemented (*n* = 19) or maybe implemented (*n* = 1) safety changes. The most common safety changes that clients had undertaken after safety coaching included preventing slip, trip, or fall hazards, clearing accessibility into and within the home, and improving the lighting in the home. Table 4 also shows that 53% of clients showed a solid sustained interest (yes, interested) in implementing safety changes at follow-up.

In post-field-study interviews, NMs and agency liaisons described the critical function of the safety handbook’s visuals and information in engaging clients. Interviewees reported the handbook as easy to read or look at, as well as informational but not overwhelming. Participants reported that the handbook helped the NMs and clients to communicate openly. The handbook provided a structure to recurring conversations: some NMs visited their clients repeatedly and discussed concerns related to home safety conditions. In one elder service agency, the liaison described this as follows:

*… [the handbook] gave more of a structure to [conversations], OK, here’s the barrier. Let’s identify and problem solve, versus just kind of going and talking to [clients] about the same things over and over again, month by month, every couple of weeks… I think all the stuff about like how to prepare for when services are coming. Which I think they, everybody was kind of the mindset like, oh, they’re coming to my house. They’re going to do whatever I need and whatever I want. And not, you have a role to do to prepare for them coming, and what’s going to make them successful for you when they’re in the home.*

- An agency liaison at the interview session #6

An NM who participated in the same above session said the following:

*I think like having [clients] see things in writing was helpful, too, because I think sometimes specifically the section where it kind of went over like the scope of work that aides can do… we have one client in particular that I was meeting with, I don’t think believed our agency or the aide’s agency when they were saying like, we can’t clean windows, and we can’t do certain things. So I think just having it in writing, outlining like the cans and cannots was very helpful.*

- An NM at the interview session #6

Another elder service agency liaison described the handbook’s facilitative role in building trust between a client and caregiver which could eventually translate into other aspects of safety:

*… what your homecare aide can and can’t do [section], I think that was huge. But then getting the most out of your homecare visit [section], like really, how to utilize, how to form that relationship with the aide, and how to really build that rapport, can be huge, because we have consumers who don’t bother to build that rapport… they might initiate arguments. They might do things like smoking… they don’t think, they’re not looked at, they view it as very transactional, as like, you’re coming in. You’re cleaning… But being able to get the most out of it and really build that relationship… if they trust their aide, and if they trust their case manager, and the RN that comes in to do their visits, they’re going to take these steps, which I think is huge.*

- An agency liaison at the interview session #4

### 3.5. Fidelity

Overall, the intervention materials and methods were implemented with good fidelity. Safety coaching was provided to HC clients at all five participating agencies as planned. This study aimed to target either new clients at each agency or existing clients who had not received recent safety education. All three HC agencies who participated in the full study were able to select new clients for safety coaching; in the modified study, two elder service agencies chose both new and existing clients. One agency (Agency #2) employed only one NM who conducted both the control and intervention arm (in that order). In the other two agencies, one NM was assigned for the intervention and one for the control arm. HC aides who evaluated the effectiveness of the intervention through home safety checklist surveys were kept blinded to the intervention in all three HC agencies. Blinding to the intervention was achieved as planned.

In one agency, fewer safety coaching baseline visits were implemented than originally planned because many of their new clients were not deemed suitable for safety coaching due to their underlying health conditions. Conducting a follow-up visit or phone call was more challenging (see Section 3.5.2).

#### 3.5.1. What Went Well during Safety Coaching and Follow-Ups?

Based on information in baseline and follow-up surveys, Table 5 presents the selected open-ended reports of NMs on the strengths of the study (i.e., what went well during baseline safety coaching and what seemed promising when following up with the client a couple of weeks afterward). The responses reflect the role of the safety handbook as a successful learning tool when engaging the clients during coaching and promising outcomes during the follow-up. The NM reports also show the importance of clients’ family support.

The post-field-study interviews supported the findings of Table 5. An agency liaison stated that successful safety coaching depended on NMs’ motivation and capacity to establish connections with their clients. When nurses show enthusiasm and explain concepts clearly and appropriately in depth, clients connect to them. The coaching process allowed NMs to engage clients with topics outside of their clinical area expertise “*through a more varied conversation*” than their usual communications. Two NMs reflected on the successful learning process of clients during safety coaching as follows:

*I was pleasantly surprised with the clients who were actually very interested in going through the booklet. I think each client learned a lot, I don’t think they realized that they also had a responsibility, and like when they have aides coming into their homes. I think they learned about kind of how to treat aides, be a little bit more respectful.*

- An NM at the interview session #6

*Our population are obviously elders, I really do think they were open to learning. A lot of them were very happy with the [safety handbook]… the images are really good to have, because some people are more visual learners. Some people do read. Whereas you targeted both in here. So I feel like when I went into the homes, you could tell the people [who] really wanted to look at the images first… depending on where their cognitive status is, even if they were slightly confused, I think the images really helped them in that case… They really wanted their homes safe, for the most part, a majority of them do. And I feel like they really like to have that information of how they can do so.*

- An NM at the interview session #3

#### 3.5.2. Challenges during Study Implementation

There were some notable challenges that affected the safety coaching and the follow-up sessions. A few clients were resistant to any change; consequently, they did not express interest in the safety topics. Clients might have been available for safety coaching; however, they were not reachable for the follow-up visit or phone call. Clients’ medical conditions—for example, frequent hospitalizations, anxiety, or memory problems—were among the reported barriers to follow-ups. In some cases, the homes were already free from clutter and had good accessibility for client and aide care tasks; therefore, no obvious safety changes were needed. Table 6 lists some of the challenges recorded during the baseline safety coaching and their follow-ups.

The post-field-study interview sessions supported the findings in Table 6. In one agency, three clients (out of five) were not available for follow-up. The NM explained that the main reason was the clients’ resistance to change:

*Some [clients] were good and were willing to hear the education of the booklet and go over it, and there were a couple that did some minor changes. But overall, people didn’t want to change. They were just kind of set in the ways of doing things, didn’t want to look at it like that. They were looking for more [than] just the home care [services], didn’t want to implement things within their [home]… they were appreciative, but didn’t want to make changes.*

- An intervention NM at the interview session #5

Another NM described how the time around the hospital or other health facility discharge can be challenging both for clients and their families to incorporate education like safety coaching:

*… even families are very overwhelmed at that [discharge] point in time. They try to keep a list of who’s with who. You know, they have the nurse from the visiting nurses. They have the nurse from us doing the initial assessment. So it’s a lot to keep on their mind and keep it organized. But again, some of these that I incorporated this field study with were not initial assessments, and that’s when we’re going in. Usually we go in yearly here as the nurse. They were able to kind of be more focused.*

- An NM at the interview session #6

### 3.6. Intervention Applicability in the HC Industry

The post-field-study interview sessions reported findings on the potential applicability of the safety coaching approach in the HC industry in general and how safety coaching resources could contribute to agency policies and practices to improve safety in various contexts. We extracted the main coded themes and subthemes (Table 7) related to the safety coaching intervention impact on the (i) HC industry and (ii) HC agency policy or practice.

An elder service agency liaison thought that every HC client could benefit by receiving a hard copy of the safety handbook or by being shown some of the home safety examples from the handbook. Nevertheless, when targeting clients who are completely new to HC services, home safety education could be even more effective. An NM in one of the participating HC agencies explained that even though the clients who received safety coaching during this study were new clients at their agency, it did not necessarily mean that they were completely new to HC services. In addition, she also reiterated the essential role of family members as primary targets. She described this as follows:

*… we have consumers in their late 80 s and their 90 s, and they’ve had homecare for years. So I feel like it may have been more effective if this was their first time having homecare in their home, rather than having homecare in their home for the past five years, and now we’re like, this is how you do this… I think that maybe not consumers themselves, but I think that a lot of family members of consumers are always looking for information, and when people get to a point where their parents need care in the home, it can be very overwhelming for them… having that material out there for [family members] to have and to see and to read can be very helpful for them.*

- An NM at the interview session #5

Fall prevention training for staff who engage with HC clients is a high priority in Massachusetts. One agency liaison explained that the study’s intervention materials, in particular the safety handbook and MI-training, could be integrated with fall prevention and other safety education activities in a friendly way so that clients do not feel like they are “being lectured at”.

The elder service agency liaisons pointed out that the safety handbook and other coaching materials would also be important resources for protective services workers. In Massachusetts, the general law pertaining to the Department of Elder Affairs (Chapter 19A) defines “protective services” in Section 14 as “services which are necessary to prevent, eliminate or remedy the effects of abuse to an elderly person“ [61]. Older adults who are “protected persons” may experience challenging life situations, for example, periodic interruption of their HC services. The handbook-facilitated safety coaching could be successful with these clients:

*But even if they have it as a resource to give out, or to, you know, use their protective service workers, using simple phrases from this book can be really helpful to convey that they are trying to help, as opposed to being like, hey, we heard that this is going on… it can be a good resource, too, of like, oh, one of those things where if you have a case conference with a protective service worker… if you state it like, hey, did you go over the safe homecare with them?… you’re able to kind of compound it into one booklet… I went over this. They’re aware of it.*

- An agency liaison at the interview session #4

A HC agency liaison surmised that the safety handbook combined with safety coaching could be a strategy to try to alleviate the turnover and “clogging of services” by setting a foundation for realistic expectations and ground rules:

*I think if you do set the foundation, and you know, because I see this a lot where there’s turnover, right. So, for example, they have a long list of people looking for services. Sometimes on that list are people who’ve gone through multiple agencies and the question is why? Well, they weren’t happy with the agencies they had. Why?… maybe if the foundation had been set right from the beginning, and they knew what to expect from the services, there wouldn’t be that long list of people waiting for services. Perhaps it could alleviate problems from the very beginning. And alleviate some of that clogging of the services if people knew what to expect right from the very beginning.*

- An agency liaison at the interview session #1

Clients also need consistency to motivate them to maintain safe conditions and continue implementing safety changes. Therefore, the consistency of training and educational techniques at each agency is important and could help ameliorate the staffing shortages. An NM described this as follows:

*Just on the point of consistency, like among staff as well, so typically if someone is in [HC program name], the goal is to transfer them to a homecare case manager. So having those same conversations, same boundaries, same accountability coming from [the program manager] as would come from a homecare case manager would come from anyone that the client was working with in the community… we’ve had like a massive staffing shortage of people being aides… I think if it was well-known that safety was being set up with clients in the home before aides went out, there might be more people willing to go into people’s homes, and that might impact the kind of staffing shortages we’ve seen across the nation.*

- An NM at the interview session #6

## 4. Discussion

### 4.1. How Did this Study Meet the Objectives, Research Questions, and Hypotheses?

This paper described the process evaluation of a safety coaching proof-of-concept intervention study designed to improve the safety of HC aides, other home-based caregivers, and HC clients. NMs used MI techniques facilitated by the safety handbook Preparing Your Home for Safe Home Care and companion video in coaching HC clients to make safety improvements in their homes. The goals of the process evaluation were to determine whether the intervention was implemented as designed and assess its strengths and challenges.

As its specific objectives, this study assessed four intervention process evaluation elements: dose delivered, dose received, fidelity, and reach. The delivered dose efficiency was 85% across all participating agencies as measured by the distribution of safety handbook copies (Table 2). For the dose received, the findings show that about 94% of clients were engaged during the baseline safety coaching; and 83% of clients who were available for follow-up showed sustained interest in implementing safety changes (Table 4). This study was implemented with good fidelity; NMs were able to document strengths (Table 5) and challenges (Table 6) during the implementation. The reach of this proof-of-concept study was 0.05%, as measured by the Massachusetts priority population who received ADLs or IADLs in 2022 [60].

To answer the process evaluation research questions, the results show that the NMs using MI techniques and safety handbook were able to (i) engage most clients during the safety coaching baseline visits and (ii) follow-up with most clients after coaching. However, not all clients engaged or responded to follow-up. NMs were able to report what worked and what didn’t work when completing the safety coaching baseline and follow-up surveys and further interpret these reports during the in-depth interviews. The process evaluation study hypothesis was confirmed: there is now mixed-methods evidence to show the strengths and challenges of the intervention implementation, in particular, how clients engaged and responded to safety coaching (Table 2, Table 3, Table 4, Table 5 and Table 6). The process evaluation in-depth interviews also contributed valuable insights to the overall feasibility of using the intervention in other populations and its impact on the HC industry (Table 7).

The video designed to complement the handbook was challenging to use during safety coaching (Table 3). A smartphone screen was too small to be useful and tablet-size screens were largely unavailable. Furthermore, the NMs regarded the safety handbook as more client-engaging than the video. This study shows that written, carefully designed resources distributed as hard copies are essential for the learning process. Providing information in two formats—(i) illustrations with short captions and (ii) text-based information—is important to support both visual learners and those who prefer to read text.

The overall intervention project aimed to assess whether the safety coaching intervention could motivate HC clients to improve safety conditions in their homes. The overall project demonstrated the effectiveness of the safety coaching intervention [39,59]: the NMs assessed that 63% of the coached clients had implemented safety changes and that almost three-quarters of the followed up clients would either continue or maybe continue to implement safety changes in the future. The overall study hypothesis was confirmed: there is now evidence that many clients would improve safety conditions in their homes if properly coached by nurses.

### 4.2. Application of Study Findings in Different Contexts

The overall intervention project followed the three-phase exploratory sequential design [47] that facilitated the r2p [48] approach. It was designed in collaboration with participating HC agencies and other HC stakeholders, including their constructive critiques of the safety handbook.

Post-field-study interview sessions offered insights about the wider applicability of the safety coaching intervention in the HC industry in general and ways in which it can contribute to safety policies and practices (Table 7). It can be applied both for staff and client training. Nurses and care managers can deploy safety coaching; in addition, protective services employees were proposed as a new group to implement safety coaching. For clients and their families, systematic coaching can contribute to safety by reducing injury and hospitalizations, particularly due to falls that may injure not only the client but also a family caregiver assisting with mobilization. Likewise, many of the points in the safety handbook are good practical strategies that improve overall home safety for residents and visitors and facilitate family members’ interactions with HC services.

### 4.3. Addressing Both HC Caregiver and Client Safety

The safety handbook was well received by clients, NMs, and participating agencies. It supported safety improvements by most of the coached clients. The handbook provided a foundation by setting realistic expectations about HC services for clients, a consistent structure for client–caregiver communications, and a facilitative role in building trust between a client and caregiver that may then contribute to other safety improvements. The safety handbook’s visuals were reported as appealing and facilitative in the client’s engagement in the safety coaching process. The safety handbook provided user-friendly, comprehensive information addressing both physical and psychological safety aspects. The safety handbook’s sections, “What Your Homecare Aide Can and Cannot Do”, and “Getting the Most Out of Your Home Care Visit”, helped clients recognize that they have a role in the success of HC services and creating safe home–work environments.

Interventions that address the safety of both caregivers and care recipients—such as the safety coaching approach reported in this paper—can influence HC staff job satisfaction and retention [15]. The findings of this process evaluation study suggest that the consistency of HC agency safety messaging and communication is significant for caregivers; therefore, a strategy—such as safety coaching—could help alleviate staffing shortages and the “clogging of HC services”. The findings of this study show that a client’s family can also be a source of strength, and therefore should be considered a key target group for safety coaching.

Our safety coaching methods and tools can complement and expand existing programs for improving HC safety and employee retention [9,10,11,12,13,35,36,37,38,62]. Flannery presented the creation of an HC coaching culture at the organizational level to improve relationships between supervisors and HC aides, and ultimately, to improve aide retention [62]. The NIOSH Total Worker Health (TWH) program approach has been used to design an HC safety and health training program that has been evaluated by randomized control trials [35,36,63]. The TWH program found promising work safety and health outcomes for HC aides (e.g., increased application of ergonomics, more effective safety communication with clients, hazard reductions in clients’ homes), and the findings were supported by HC clients’ reports on the improvements of aides’ safety behavior [35,36]. Safety checklist approaches have been applied to address HC safety hazards. As a risk identification tool, Gershon and colleagues designed a 50-item household safety checklist and an accompanying training program to improve safety and quality in home healthcare [38].

### 4.4. Limitations of this Study

As presented in the Results Section 3.5.2 (Table 6) and elsewhere, there were various challenges during this study’s implementation. These included clients’ resistance to change and health conditions that prevented participation in the safety coaching follow-up, either in person or by phone. The video designed to complement the handbook was challenging to use with a smartphone and would require at least a tablet-sized screen; MNs also found the safety handbook to be a more client-engaging tool than the video.

The different NMs who implemented safety coaching had different engagement and communication styles with clients.

The sample sizes were small, and therefore, we could not show statistically significant power for quantitative results (Table 4). Agencies selected the new clients for safety coaching, and the identities of participating clients were not disclosed to the research team. For this reason, the target population that received safety coaching cannot be generalized to the older adult population across the United States.

## 5. Conclusions

The nurse-led MI-based coaching intervention facilitated by the safety handbook was largely implemented as planned. It engaged HC clients during their initial HC assessment and supported them in creating safer home conditions. It has the potential to provide a consistent structure for caregiver–client communications and building trust between a caregiver and client. The safety coaching can set a foundation for realistic expectations about HC services among clients and their families from the start and help them recognize that they have an important role in creating successful and safe HC service visits.

The intervention described here has the potential to be scaled up and evaluated in a larger population by incorporating it into a wide range of safety education activities at HC agencies. Safety coaching could be implemented in the context of any new client intake visit. Existing clients who have not received any recent safety communication can also benefit from coaching. In addition to nurses and care managers, other HC staff such as protective services employees could be trained to implement the safety coaching.

## Figures and Tables

**Table 1:** Demographic characteristics of NMs (*n* = 7) and agency liaisons (*n* = 5) who completed and returned the anonymous demographic survey. One participant did not return the survey.

Demographic Variable	NMs/Liaisons (*n* = 12)
Gender (female) *n* (%)	11 (91.7%)
Age mean (SD)	44.3 (11.6)
Race and ethnicity	
White *n* (%)	11(91.7%)
More than one race *n* (%)	1 (8.3%)
Hispanic or Latino *n* (%)	0 (0%)

**Table 2:** The key intervention activities and materials distributed at different participating agencies.

Agency	Intervention Activity				
	Number of Safety Coaching Intervention Packets * Distributed to Agencies to Train NMs	Number of Online MI-Training Courses Completed by NMs	Number of Safety Handbooks Distributed to Agencies for Safety Coaching	Number of Safety Handbooks Given In-Person to HC Clients by NMs during Coaching	% of Safety Handbooks Distributed to HC Clients— the Efficiency of Key Dose Delivered
Agency 1	1	1	8	5	63%
Agency 2	1	1	10	10	100%
Agency 3	1	1	6	4	67%
Agency 4	1	1	6	5	83%
Agency 5	2	2	10	10	100%
Total all:	6	6	40	34	85%

* An intervention packet included the following key resources: the online MI-training course, video, safety coaching resource binder for NMs comprising the safety handbook, supplemental MI-based coaching tools, baseline and follow-up online survey questions, follow-up script/note recording tool, tracking documents.

**Table 3:** Engagement of clients during the safety coaching visits (*n* = 34) by nurse managers (NMs).

Safety Coaching Engagement Type by NMs	Number of Safety Coaching Visits (%)
Show the client the safety handbook	34 (100)
Discuss the safety handbook sections with the client while using motivational interviewing	32 (94)
Leave the safety handbook hardcopy with the client/family	34 (100)
Show the client the video that accompanied the safety handbook	1 (3)
Let the client know about the safety coaching follow-up communication in 1–2 weeks	34 (100)

**Table 4:** Assessment of clients’ engagement levels during the safety coaching baseline visits (*n* = 34) and sustained client interest levels in implementing safety changes during the follow-ups (*n* = 30) by nurse managers (NMs).

Safety Coaching Baseline Visits (*n* = 34)		
Engagement Level at Baseline	Number of Safety Coaching Baseline Visits (%)	Examples of Open-Ended Comments about the Clients’ Engagement by NMs
Yes, interested	17 (50)	The client’s son was present as well and stated the client can be stubborn regarding changes. [The client] was engaged when reviewing.
Maybe interested	15 (44)	Client was engaged, but tends to get off topic, or speak about how he does everything correctly already. [Nurse] was able to redirect the client and continue to engage him.
No, not interested	2 (6)	No comments provided
**Safety Coaching Follow-Up Visits (*n* = 30)**		
**Sustained Interest at Follow-Up**	**Number of Safety Coaching Follow-Up Visits (%)**	**Examples of Open-Ended Answers in** **the Follow-Up Survey by NMs**
Yes, interested	16 (53)	The client explained ways in which she is making changes.
Maybe interested	9 (30)	Client seems interested in implementing changes, but his mental health/behaviors may inhibit him from doing so.
No, not interested	5 (17)	When asked if the client was able to review the booklet she reported “I glanced at it, didn’t get to sit down and read it… too much going on”.

**Table 5:** Examples of open-ended survey reports by nurse managers (NMs) at each participating agency on aspects that went well during the safety coaching baseline visit and what seemed promising during the safety coaching follow-up. The illustrated reports do not necessarily refer to the same client.

Open-Ended Survey Responses by NMs	What Went Well during the Safety Coaching Baseline Visit?	What Seemed Promising during the Follow-Up Visit or Phone Call—About 1–2 Weeks after the Safety Coaching?
NM at agency 1	Client has good support system who is engaged and motivated to do what is best for the client.	Client actually implemented suggested changes (e.g., grab bar, bedside commode)
NM at agency 2	Client asked questions and continued to verbalize [understanding] of teaching.	Client stated wanting to follow recommendations on home safety to prevent falls.
NM at agency 3	Client was able to sit through the review of the booklet, and he engaged with the motivational interviewing.	Client remembered what was discussed during safety coaching… reported making changes, such as opening the curtains to allow for more light.
NM at agency 4	The client was alert/oriented and willing to listen to my coaching. She has a supportive husband as well.	… she gave examples of what she was doing (e.g., putting her cat in a closed room, working on clutter in common area, and trying to be prepared with cleaning supplies).
NM-A at agency 5	Excellent booklet [safety handbook] with visuals and written information which the client found helpful.	Client [is] very interested in booklet/information. Reported by client/family “We already knew a lot of that stuff but it was helpful”
NM-B at agency 5	The daughter was happy regarding the information as it seems the client is now willing to make some changes in the home to allow for more room in her bathroom to be able to easily get in and out with her walker.	The client was open to any changes needed/continuously needed to create a safe home environment. Particularly client has had disability/needed handicap accommodations for a while, therefore has learned to live with needs.

**Table 6:** Examples of open-ended survey reports by nurse managers (NMs) on challenges during the baseline safety coaching and follow-up visits. The illustrated reports do not necessarily refer to the same client.

Open-Ended Survey Responses by NM	Challenges during the Baseline Safety Coaching	Challenges during the Follow-Up of the Safety Coaching
NM at agency 1	Client was more concerned about pharmacy changes [not safety].	Client is anxious and can become overwhelmed easily if given too much information at one time.
NM at agency 2	Client lives with family members that don’t follow home safety making it unsafe for the client.	Client appeared overwhelmed with information.
NM at agency 3	Elder has a history of hoarding, and while she understands that it can be a problem, she has a difficult time getting rid of belongings.	[Client] continues to have frequent hospitalizations. Maintaining his home environment and consistent service is difficult for him due to his health concerns.
NM at agency 4	The client had no interest in my explanation of the book.	Client just was not interested.
NM-A at agency 5	Client’s resistance to care.	Client reported “my memory isn’t that good” and reported it was her birthday on date of call but that she did not remember the booklet.
NM-B at agency 5	The client’s attention was scattered at times as she had recently been discharged from the hospital.	This client was challenging due to her being set in her ways and reporting “I like things done my way”.

**Table 7:** Main themes and subthemes on the potential impact of safety coaching intervention on the HC industry in general, as well as on policies and practices at HC agencies as reported in six post-field-study interview sessions.

Themes	Subthemes
**Impact on the HC** **industry**	• Protective service workers are an important audience for the handbook
	• Safety coaching can be deployed by both nurses and care managers
	• Training for both staff and clients
	• Setting the foundation before the HC services start for the client
	○ Target clients who are completely new to HC services
	○ Clients with challenges may have gone through multiple agencies
	• Consistency is key for clients and staff
	○ Hearing the same messages from multiple skilled HC services
	○ Different staff members have the same safety conversations with clients
	• Coaching contributes to keeping clients safe at home
	○ Avoiding hospitalizations
	○ Family members of clients need information
	• Could address HC staffing shortages and alleviate turnover rates
**Impact on the** **HC agency** **policy or** **practice**	• Introduce the handbook and coaching resources to the protective service team
	• Incorporate the handbook for both new client and new caregiver orientations
	• Both elder service and HC agencies give the handbook to clients
	○ Include the handbook in the new client folders in the future
	• Consider as best practices approach for HC worker and client safety
	• Can be integrated into many safety education/communication activities
	○ For example, fall prevention training

## Data Availability

The data for this study are confidential as required by the IRB approvals. To protect the anonymity of the participants, the data are not publicly available. Information about the data, methods, and the study in general can be requested from the corresponding author.

## Appendix A

Examples of questions asked in the post-field-study interview sessions. Examples of interview session questions reported in this study.

(1) Tell us your first name, your job in the home health care, and how long have you been working in this industry?
(2) Did you have a chance to go over the field study summary? Please share any findings/information that surprised you?
(3) Your agency [NAME OF THE AGENCY] completed the field study [DATE OF STUDY COMPLETION]. Please share any broad views how you feel the field study went overall.
  - What went well?
  - What was challenging?
  - Did HC aides pass any feedback to you about the study that would be helpful for us to know?
  - The Safe Home Care Project prepared resource binders for both the control and intervention paths. How helpful were these binders when you conducted the study?
  - These 4 materials/resources were part of the study’s safety coaching package, how helpful were these (not helpful/somewhat helpful/very helpful)
    1. The Preparing Your Home for Safe Home Care booklet?
    2. The Motivational Interviewing (MI) online training?
    3. The optional video accompanying the booklet?
    4. The optional online OSH training?
  - Did the study safety coaching package contribute to generalized safety awareness among consumers? Please share your thoughts.
(4) If your agency completed the control path before starting the intervention path. How did you select clients for the control path?
  - Did any control client ever find out that HC aides were conducting safety/hazard surveys for their homes? If yes, please describe any reactions from these clients.
(5) How did you select clients for the safety coaching intervention path?
  - Did any HC aides who completed safety/hazard surveys for the intervention homes find out about the intervention status of the client? If yes, please describe any reactions from these HC aides.
  - Did any HC aides report to you seeing the booklet at the client’s home? If yes, how did they describe this to you?
(6) The intervention path started with the Motivational Interviewing (MI) Training Program. How was your MI Training experience in general?
  - How did MI Training help you to safety coach the intervention clients?
  - How did MI Training complement the booklet?
(7) Please describe your safety coaching process for the intervention clients. You can be as broad or specific as you like.
  - How did the intervention clients react when you showed the booklet?
  - Did you ever use the video for the clients? If not, why? If yes, how did the clients react to the video?
(8) For the clients’ engagement level, the baseline survey results showed that in your agency [NUMBER OF CLIENTS] were maybe interested and [NUMBER OF CLIENTS] were interested when you coached with the booklet. Please share any specific signs showing the clients’ interest at this baseline stage.
(9) For the client follow-up, we developed a sample follow-up script/note recording tool that was included in the binder. How helpful was this tool to follow up with the client?
  - Your agency reported following up [NUMBER OF CLIENTS] of the intervention clients by phone and with [NUMBER OF CLIENTS] in-person. What were pros and cons in each follow-up method? How did you decide whether to follow-up by phone or in-person? To what extent did the COVID pandemic affect the type of the follow-up method?
  - Were there any other aspects of this study that you think were impacted by COVID? Please explain.
  - How did you feel about the timing of the follow-up after your safety coaching? Was it convenient, too soon, or too late?
(10) At the follow-up, your agency indicated that [NUMBER OF CLIENTS] were still interested in making home safety changes and [NUMBER OF CLIENTS] maybe interested in making home safety changes. Please share any specific signs that made you assess the clients’ interest at the follow-up stage.
(11) Your agency reported that most home safety changes that occurred after the safety coaching addressed [NAMES OF SPECIFIC HAZARDS]. Was this surprising to you?
(12) Now when we have completed the field study at your agency, was it worth your time to participate? Please explain.
  - How has this field study and its resources impacted your agency policy and/or practice?
  - Is your agency planning to use any of the field study resources—the booklet, online video, online MI training, or online OSH training—in educational or training activities?
  - How could this field study and its resources impact the HC industry as a whole? In addition to the booklet and other resources, what do clients need to motivate them to make safety changes in their homes?
(13) If we were to design a similar study in the future, what would you recommend we do differently?
(14) We wanted you to (i) help us interpret selected field study findings, (ii) offer feedback on different field study procedures and steps, and (iii) have you tell us how the field study influenced your workplace policy or practice. Is there anything that we missed or anything else that you want to share with us?
